# Crystal structure of the 5hmC specific endonuclease PvuRts1I

**DOI:** 10.1093/nar/gku186

**Published:** 2014-03-14

**Authors:** Asgar Abbas Kazrani, Monika Kowalska, Honorata Czapinska, Matthias Bochtler

**Affiliations:** 1 International Institute of Molecular and Cell Biology, Trojdena 4, 02109 Warsaw, Poland; 2 Institute of Biochemistry and Biophysics, Polish Academy of Sciences, Pawinskiego 5a, 02106 Warsaw, Poland

## Abstract

PvuRts1I is a prototype for a larger family of restriction endonucleases that cleave DNA containing 5-hydroxymethylcytosine (5hmC) or 5-glucosylhydroxymethylcytosine (5ghmC), but not 5-methylcytosine (5mC) or cytosine. Here, we report a crystal structure of the enzyme at 2.35 Å resolution. Although the protein has been crystallized in the absence of DNA, the structure is very informative. It shows that PvuRts1I consists of an N-terminal, atypical PD-(D/E)XK catalytic domain and a C-terminal SRA domain that might accommodate a flipped 5hmC or 5ghmC base. Changes to predicted catalytic residues of the PD-(D/E)XK domain or to the putative pocket for a flipped base abolish catalytic activity. Surprisingly, fluorescence changes indicative of base flipping are not observed when PvuRts1I is added to DNA substrates containing pyrrolocytosine in place of 5hmC (5ghmC). Despite this caveat, the structure suggests a model for PvuRts1I activity and presents opportunities for protein engineering to alter the enzyme properties for biotechnological applications.

## INTRODUCTION

The mechanistic basis of modification specific DNA binding and - in some cases - cleavage has attracted much interest. Based on experimental structures or confident homology models, we now have a detailed picture of 5-methylcytosine (5mC) specific binding by MBD1 (1), MBD2 (2), MBD4 (3), MeCP2 (4), Kaiso (5) and the replication fork-associated UHRF1 (6–8). There are also structural data about 5mC specific enzymes: McrBC has been crystallized in complex with DNA (9), and for MspJI a very informative structure in the absence of DNA has been determined (10). Based on these studies, the methyl binding proteins/enzymes can be divided into two broad groups, depending on whether they recognize the methyl group in the context of double stranded DNA or whether they flip the modified base to scrutinize it in a dedicated pocket. MBDs (1–4), MeCP2 (4) and Kaiso (5) interact with the modified base in a Watson-Crick pair. In contrast, UHRF1 and most likely also MspJI share a so-called SRA (SET and RING associated) domain that flips and accommodates the modified base (6–8,10). The same is true for McrBC, even though the flipped base binding domain is in this case not homologous to the SRA of UHRF1 and MspJI (9).

The presence of 5-hydroxymethylcytosine (5hmC) in phage (11) and mammalian DNA has been known for a long time (although the initial estimates for the amount of 5hmC in mammalian DNA were too high). Much recent research was triggered by the identification of the function of TET (ten-eleven translocation) proteins as 5mC oxidizing enzymes (12–13), and the role of 5hmC as a demethylation intermediate (14–15), epigenetic mark (16) and diagnostic marker in cancer (17). Recent pull-down/mass spectrometry studies have also shown that there is a large repertoire of 5hmC binding proteins in vertebrate tissues (18–19). Some 5hmC binding proteins, such as UHRF1, bind also 5mC, and their interaction with 5hmC can be modeled based on the interactions with 5mC (20). Other proteins, such as MBD3, which binds to 5hmC according to some (21) (but not other (18)) studies, are homologous to structurally characterized 5mC binding proteins and therefore their possible interactions with 5hmC can be deduced (21). However, for most other proteins that were identified in the mass spectrometry experiments, it is not even clear whether the interaction with 5hmC is direct, and provided it is, how the 5hmC base is ‘read’. It is also still not understood how the presence of the 5hmC base can trigger an enzymatic reaction.

The endonuclease PvuRts1I from *Proteus vulgaris* (strain) has been reported to be a dimer (22) like most endonucleases that catalyze double strand breaks. It cleaves DNA that contains either 5hmC or 5ghmC (5-glucosylhydroxymethylcytosine) bases (23), with a preference for α-5ghmC over β-5ghmC (22). Cleavage is most efficient when two 5hmC (5ghmC) bases are present in opposite DNA strands approximately 22 bases apart from each other (24). PvuRts1I makes a double strand break approximately in the middle between the two sites (the precise pattern is 5′-*C*N_11–13_↓N_9–10_G-3′, where the arrow denotes the cleavage site and *C* the modified base) (24). Some double strand cleavage can also be observed when there is only a single 5hmC (5ghmC). The potential applications of 5hmC sensitive sequencing have triggered the search for PvuRts1I homologs that exhibit desirable properties for biotechnological use. This search has led to the identification of a whole family of enzymes, which differ slightly in the distance requirement for the modified bases (25). In contrast to PvuRts1I, some of them such as AbaSI show a preference for 5ghmC over 5hmC (25), which can be exploited in sequencing by postglucosylation of 5hmC with phage T4 glucosyltransferase. All tested members of the PvuRts1I family discriminate between 5hmC and 5mC, but to varying degrees. As 5hmC is much rarer than 5mC in animal genomes (26), very high discrimination stringency is required for biotechnological use. Hence, there are at least two engineering goals to improve PvuRts1I and/or the other family members. First, it would be desirable to design an enzyme fully dependent on a single modified site only, which should make a double strand break on one or both sides of the modified base. Second, it would be useful to improve the stringency of 5hmC versus 5mC discrimination.

Here, we report the crystal structure of PvuRts1I at 2.35 Å resolution. The structure reveals an N-terminal PD-(D/E)XK domain in agreement with an earlier prediction (27) and a previously unrecognized C-terminal SRA domain. Site-directed mutagenesis experiments confirm the importance of predicted key residues in the structure. Based on the combined crystallographic and biochemical data, we suggest a structural explanation for why PvuRts1I requires 5hmC or 5ghmC bases in opposite strands at a distance of just over 20 base pairs for the introduction of a double strand break approximately halfway between the modified bases.

## MATERIALS AND METHODS

### Cloning

A codon optimized PvuRts1I REase (*pvuRts1I*) synthetic gene in pTriEx (Ap^r^) vector was purchased from Mr. Gene (Germany). The gene was introduced into pET15bmod (Ap^r^), a derivative of pET15b (+) (Ap^r^) via EcoRI and XhoI restriction sites, resulting in a construct coding for the protein with N-terminal MGHHHHHHEF tag. The same gene was also cloned into pET28a (+) (Kn^r^) via NcoI and XhoI, leading to a construct for a protein with slightly modified N-terminus (MGSK…) and C-terminal tag (LEHHHHHH). Mutants of *pvuRts1I* were generated in the construct for the N-terminally tagged protein variant using the QuikChange protocol (28).

### Protein expression

Expression experiments were done in *Escherichia coli* strain ER2566 (F- λ- fhuA2 [lon] ompT lacZ::T7 gene 1 gal sulA11 Δ(mcrC-mrr)114::IS10 R(mcr-73::miniTn10-TetS)2 R(zgb-210::Tn10)(TetS) endA1 [dcm]) (from New England Biolabs). The strain was transformed with plasmids coding for the N- or C-terminally tagged versions of the PvuRts1I protein. Cells were grown in LB medium with 50 μg/ml ampicillin at 37°C to OD_600_ of 0.6 and induced with between 0.1 and 1 mM IPTG. The expression was highest when cells were grown for 4 h at 22°C. Cells were harvested by centrifugation and the pellet was stored at -20°C. Expression of the selenomethionine version of PvuRts1I (with N-terminal tag) was done in methionine auxotrophic BL834(DE3) cells in defined media lacking methionine and supplemented with selenomethionine (29).

### Protein purification

Frozen cells expressing PvuRts1I were thawed and resuspended in buffer A (20 mM Tris/HCl pH 7.6, 400 mM NaCl and 1 mM PMSF). Cells in suspension were opened by sonication and the cell debris was removed by centrifugation at 145000×g for 30 min. PvuRts1I was purified by affinity chromatography on nickel nitrilotriacetic acid (Ni-NTA) agarose resin (Qiagen). The protein was eluted in a gradient of imidazole (30 mM to 300 mM) in buffer B (20 mM Tris/HCl pH 7.6, 200 mM NaCl and 7 mM 2-mercaptoethanol). Fractions containing PvuRts1I were combined and concentrated using Vivaspin concentrators (10 kDa MWCO). The protein was purified further by size exclusion chromatography on HiLoad 16/60 Superdex 75 column (GE Healthcare), equilibrated with buffer C (20 mM Tris/HCl pH 7.6, 200 mM NaCl, 1 mM EDTA and 1 mM DTT). Fractions containing PvuRts1I were pooled and concentrated to 20–24 mg/ml. From 1 liter of culture, ∼7 mg of protein was obtained that appeared pure on a Coomassie-stained SDS-PAGE gel. The variant proteins were obtained according to the protocol for the wild-type.

### Crystallization

PvuRts1I was concentrated to 23 mg/ml and then the buffer was supplemented with 0.2 M glucose. Crystals were grown at 18°C by the hanging drop method. A mix of 2 μl of protein solution and 2 μl of reservoir buffer was equilibrated against reservoir buffer containing 10% w/v PEG 4000, 20% v/v glycerol, 20 mM D-glucose, 20 mM D-mannose, 20 mM D-galactose, 20 mM L-fucose, 20 mM D-xylose, 20 mM *N*-acetyl-D-glucosamine and 0.1 M MOPS/HEPES-Na pH 7.5. For cryo-protection, crystals were transferred to modified reservoir buffer supplemented with 28% instead of 20% v/v glycerol.

### Structure determination

Crystals belonged to space group P4(1)2(1)2 with cell dimensions *a* = *b* ≈ 62 Å, *c* = 211 Å and contained one molecule of PvuRts1I in the asymmetric unit. They diffracted to approximately 3 Å resolution. In the absence of suitable models for molecular replacement, experimental phasing was required. Therefore, we grew crystals of the selenomethionine variant of the protein (containing four selenium atoms not counting the initiator methionine upstream of the histidine tag), which turned out to be better than the wild-type protein crystals and diffracted to 2.35 Å resolution. Diffraction data were collected at a wavelength of 0.97625 Å, which is just above the selenium edge in energy. The structure was solved by the single anomalous diffraction (SAD) method. Selenium sites were localized using the SHELXD program (30). The SHELXE program (31) was then used to generate an experimental electron density map by a combination of phasing and density modification steps. The density was interpreted automatically using PHENIX (32), leading to a nearly complete model of PvuRts1I lacking only a few loops. The model was completed and improved manually and refined with the COOT (33) and REFMAC (34) programs (Supplementary Table S1). The final model coordinates and the corresponding structure factors were deposited at Protein Data Bank (PDB) with the 4OQ2 accession code.

### Assay of the PvuRts1I mutants against T4 phage genome

T4 phage DNA was purified according to a published protocol (35). All the mutants and the wild-type protein were loaded on the SDS gel to show the equal concentration of the proteins. Approximately 0.1 μg (low) and 1 μg (high) amounts of protein were mixed with 240 ng of T4 phage genome in the buffer containing 50 mM potassium acetate, 20 mM Tris-acetate, 10 mM magnesium acetate and 1 mM DTT, pH 7.9. The reactions were incubated at 23°C for 20 min. The reaction mixtures were loaded to 1% agarose gel and visualized by Gel Red (Biotium Inc.) staining.

## RESULTS

### PvuRts1I expression and biochemical characterization

A synthetic gene was used to overexpress versions of PvuRts1I with N-terminal or C-terminal hexahistidine tags in *E. coli* strain ER2566. The proteins were purified by affinity and size exclusion chromatographies. The purified recombinant wild-type proteins with tags on either end, but not controls with changes to important residues, were active against T4 phage DNA, which is known to contain a large number of 5ghmC bases at various distances to each other. Although protein activities were at least qualitatively in agreement with the literature data, the variant of PvuRts1I with N-terminal hexahistidine tag had some other unexpected properties, at least in our hands. While PvuRts1I should be a dimer also in the absence of DNA (22), size exclusion chromatography with the N-terminally tagged, but not the C-terminally tagged, variant of the enzyme suggested a slightly lower than expected molecular mass. We also observed a high and unspecific affinity for DNA (hydroxymethylated, methylated and non-methylated DNA are all bound) (Supplementary Figures S1 and S2). Despite these undesirable features of the N-terminally tagged PvuRts1I, we continued work with this variant of the protein, because it yielded well-diffracting crystals, at least in the absence of DNA.

### Crystallization and structure determination

Crystallization of PvuRts1I was attempted either in the absence of DNA or with oligonucleotides containing two 5hmC bases at the appropriate distance. We either avoided divalent metal cations or used Ca^2+^ ions, which support DNA binding, but not cleavage. Finally, we also set up crystallization trials with oligoduplexes that represent PvuRts1I cleavage products (except for the 5′-phosphates), in the presence of either Mg^2+^ or Ca^2+^ ions. All these experiments did not yield any diffracting crystals. We concluded that PvuRts1I might have a flexible substrate binding site, and because we knew that the enzyme accepted 5ghmC containing DNA, we tried crystallization in the presence of large amounts of glucose. This proved crucial for crystallization success. Crystals belonged to space group P4(1)2(1)2, contained one molecule of PvuRts1I in the asymmetric unit and diffracted up to 2.35 Å resolution. The structure was solved by the SAD method using a crystal of the selenomethionine version of the protein.

### Gross structure of PvuRts1I

**Figure 1.**
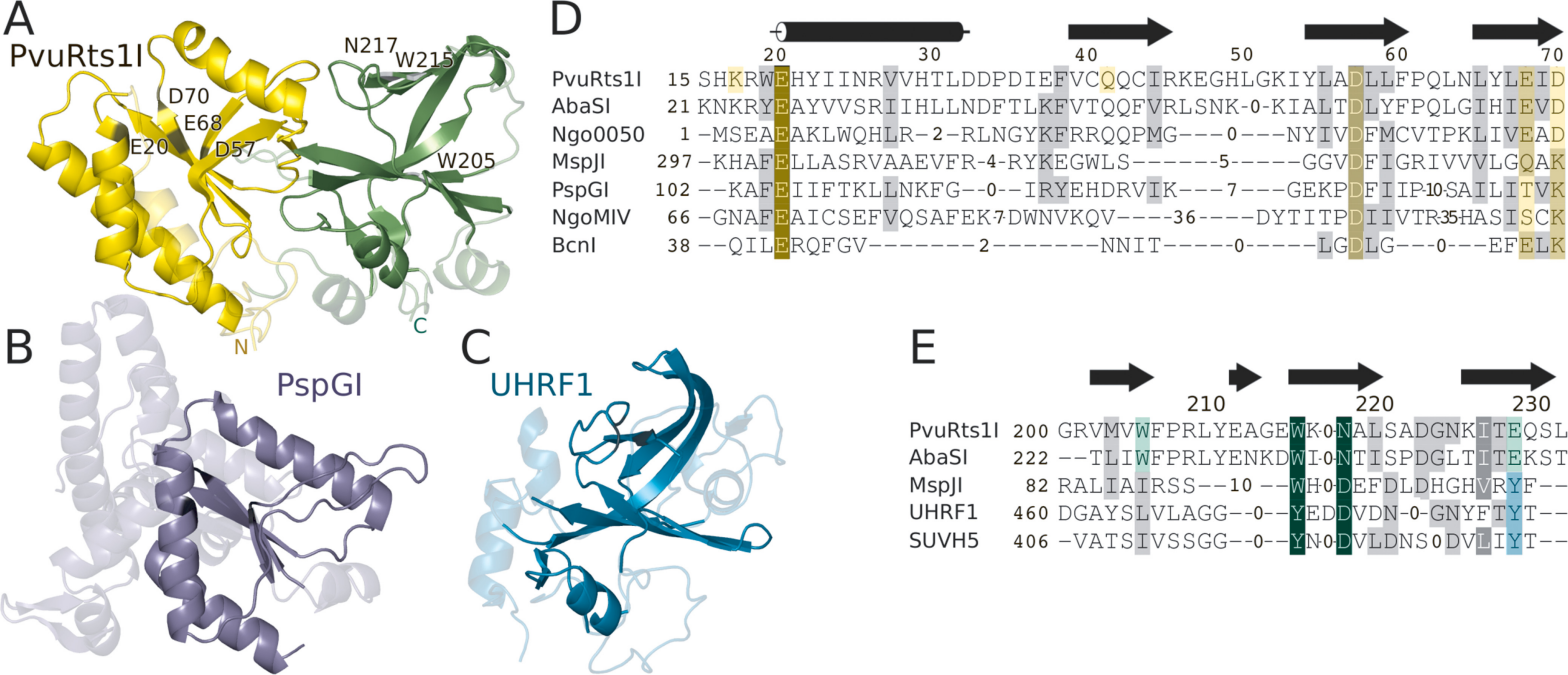
PvuRts1I domains and homologs. (A) PvuRts1I catalytic (residues 1–140) and SRA (residues 141–293) domains are in yellow and green, respectively. Core elements of the fold are in bright and additional elements in faint color. (B) PspGI restriction endonuclease, a homolog of PvuRts1I catalytic domain. (C) SRA domain of UHRF1, a homolog of PvuRts1I SRA domain (6–8). (D) Alignment of the amino acid sequences of PvuRts1I catalytic core and homologs. (E) Alignment of the SRA domain sequences in the pocket region. The PvuRts1I-AbaSI alignment is sequence based, all other alignments are structure based.

The crystal structure reveals that PvuRts1I is a two-domain protein (Figure 1). We carried out DALI searches with the two domains against the PDB database of protein structures (36). The results indicate significant structural similarity between the N-terminal domain of PvuRts1I and several PD-(D/E)XK endonucleases (DALI Z-scores of 6.9 for Ngo0050 from *Neisseria gonorrhoeae*, 4.9 for V.EcoKDcm, 4.2 for Hjc, 2.7 for PspGI and 2.5 for NgoMIV), in agreement with the inclusion of PvuRts1I in a bioinformatic survey of highly diverged PD-(D/E)XK restriction endonucleases (27). We therefore conclude that the N-terminal part of the enzyme (residues 1–140) harbors the nuclease activity and henceforth refer to it as the catalytic domain. The DALI search also revealed a previously unrecognized clear structural similarity between the C-terminal part of PvuRts1I and various SRA domain proteins (DALI Z-scores for the corresponding domains: 7.0 for SUVH5, 6.5 for UHRF1, 6.1 for MspJI and 6.0 for UHRF2). Therefore, the C-terminal domain of PvuRts1I (residues 141–293) will be referred to as its SRA domain in the following. As SRA domains recognize modified bases by flipping them out of the DNA stack into a pocket of the domain (6–8), the PvuRts1I could also be a nucleotide flipping enzyme.

### Structure of the nuclease domain and active site prediction

**Figure 2.**
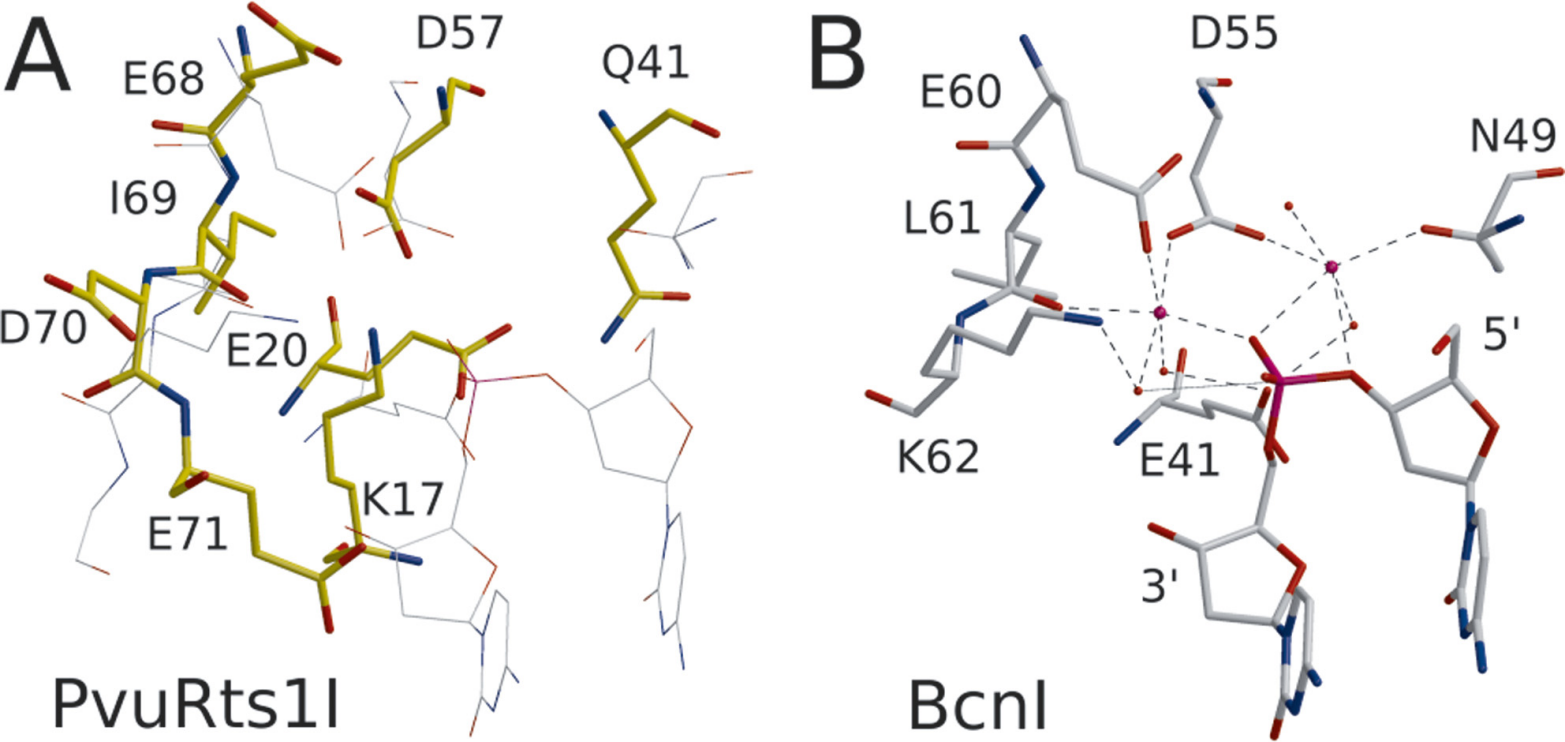
Active site. PvuRts1I (A, yellow) and BcnI as bona fide PD-(D/E)XK endonuclease with DNA (B, gray) in all-atom representation. The thin lines in the PvuRts1I panel indicate the positions of the corresponding residues and substrate DNA in the BcnI structure.

PD-(D/E)XK restriction endonucleases are named for the (typically) conserved residues in the active site, which are found in canonical secondary structure contexts. The core folding motif of PD-(D/E)XK restriction endonucleases consists of an α-helix that is followed by three consecutive β-strands, which together form an antiparallel β-sheet (often with additional strands outside the core motif) (Figure 1). The PvuRts1I catalytic domain contains the PD-(D/E)XK motif and, as predicted by the bioinformatic analysis (27), has candidate active site residues in the expected places (with the exception of the lysine) (Figure 2). The first catalytic residue, a glutamate in α-helical context that is not cited in the PD-(D/E)XK consensus, is Glu20. The ‘PD’ aspartate, which coordinates one or both metal ions in the PD-(D/E)XK family, is Asp57 (sequence context ADLL) at the N-terminal end of the second β-strand of the core motif. The canonical ‘(D/E)XK’ motif in PvuRts1I is changed to ‘EID’, with Glu68 in the role of the acidic residue of the motif and an aspartate in the place of the expected lysine residue. There is a lysine residue (Lys17) elsewhere in the sequence that is spatially in the proximity of the active site, but the ϵ-amino group is not in the expected location. Metal ions are not present in the active site because crystals were grown in the absence of divalent metal ions.

### Identification of active site residues by site-directed mutagenesis

**Figure 3.**
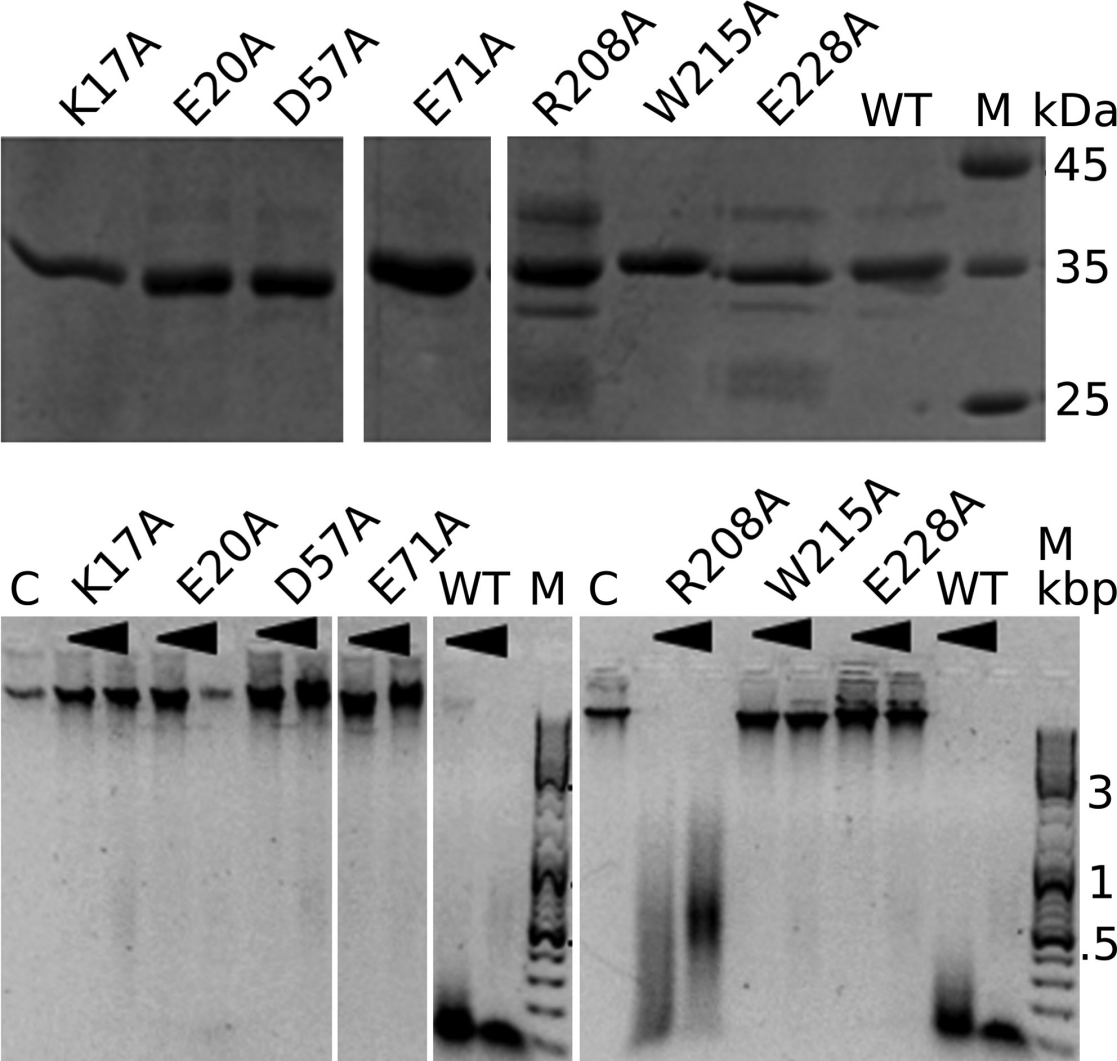
Effect of mutations on PvuRts1I activity. PvuRts1I and its variants were either analyzed for protein purity by SDS-PAGE and Coomassie staining (top, 3 μg per lane) or used to digest 240 ng of phage T4 DNA, which was then analyzed by gel electrophoresis in a 1% agarose gel and stained with Gel Red. For each variant, two protein amounts (0.1 μg, left; 1 μg, right) were tested.

Candidate catalytic residues and Glu71, an acidic residue without clear function in the PD-(D/E)XK motif, were individually replaced by alanines. The activity of the resulting variants was tested with T4 phage DNA as a substrate in conditions that lead to complete DNA cleavage by the wild-type enzyme (Figure 3). As expected, replacement of Glu20 or Asp57 with alanine strongly reduced or abolished activity. The role of the Glu68 in catalysis could not be directly tested because the Glu68Ala variant of PvuRts1I could not be made in soluble form in *E. coli*. Mutation of Glu71 also abolished activity, and at most residual activity was seen when Lys17 was mutated to alanine. We conclude that the mutagenesis experiments support the bioinformatic- (27) and crystallography-based identification of active site residues. These residues were also noted, but not singled out, in an earlier study of amino acid conservation in the PvuRts1I family (25).

### Structure of the SRA domain and model for the DNA recognition

**Figure 4.**
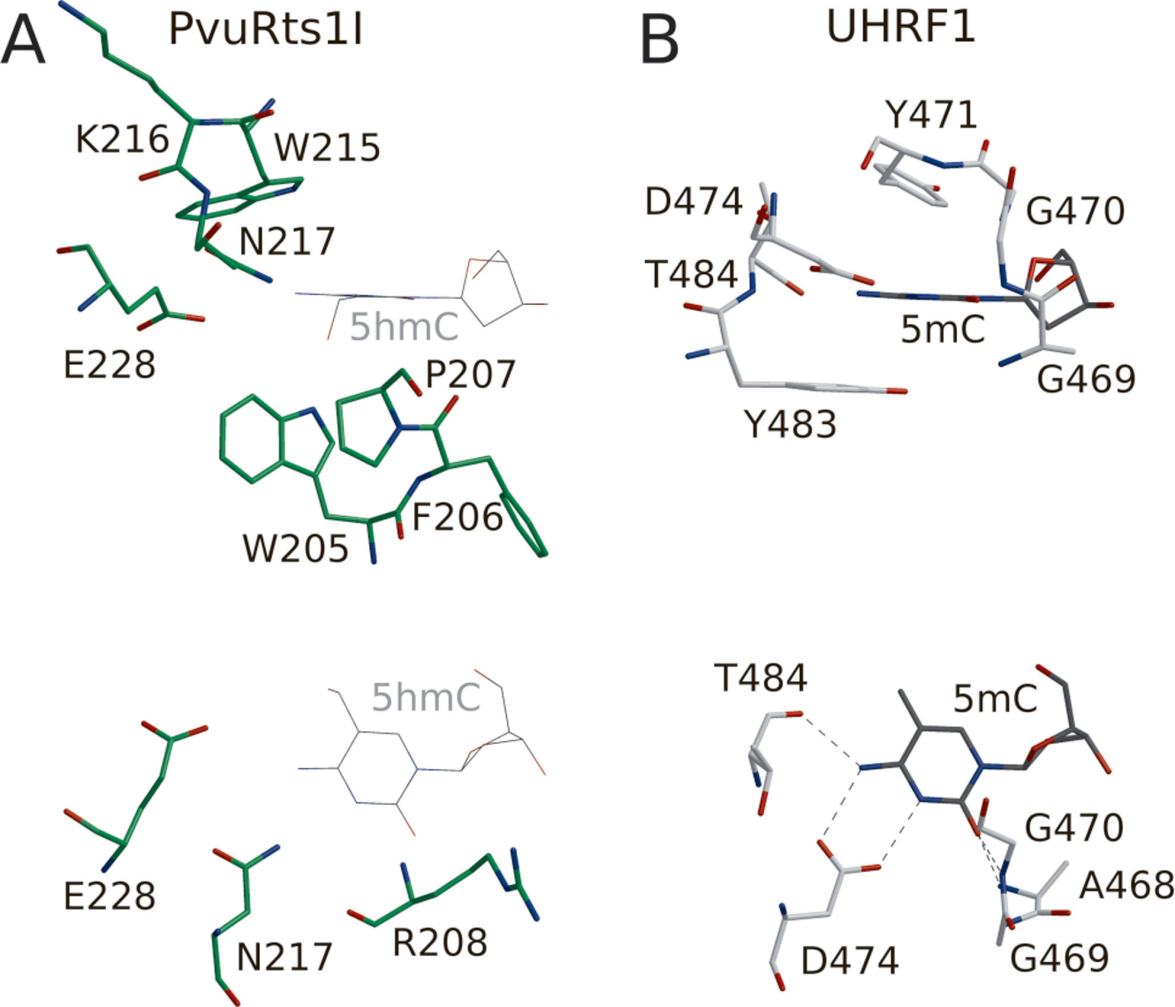
SRA domain pocket. PvuRts1I (A, green) and UHRF1 (B, gray) in all-atom representation. The flipped 5mC base in the UHRF1 pocket is observed in the crystal structure. In contrast, the 5hmC base in the PvuRts1I pocket has been modeled based on the superposition of the two structures (A, thin lines).

The C-terminal domain of PvuRts1I has the typical SRA domain fold. It is organized around a central, mixed β-sheet with additional smaller β-sheets and some helices wrapped around it. Among the SRA domains, the binding to DNA is experimentally best characterized for UHRF1 (6–8). Hence, we superimposed the SRA domain of PvuRts1I onto the UHRF1-DNA co-crystal structure and checked the location of the flipped base (Figure 4). This analysis reveals that PvuRts1I has indeed a pocket in the expected location, with sufficient space for 5hmC (Figure 4 and Supplementary Figure S3). The pocket is formed mainly by Pro207, Trp205, Trp215, Asn217 and Glu228. A potentially flipped base could make hydrophobic contacts with Trp215 from one side and Pro207 and Trp205 on the other. The 5hmC Watson–Crick edge might be recognized by Asn217, Glu228 and Arg208. The modeling also shows that there is extra space in the PvuRts1I pocket, so that even 5ghmC might fit in. Given the uncertainties of the modeling (only 13% sequence identity between the SRA domains of PvuRts1I and UHRF1), we cannot pinpoint the precise location of the hydroxyl or glycosylhydroxyl group, but Trp205, Arg244, Tyr237 and Glu228 are candidate interaction partners for hydrogen bonding. Interestingly, we see an isolated large peak of electron density close to the expected location of the glucosylhydroxymethyl group in the pocket for the flipped base. Unfortunately, the resolution of the structure is not sufficient to decide whether this peak is due to a partially disordered glucose molecule (which would explain why it was essential for crystallization).

### Tests of nucleotide flipping and the SRA pocket function

The detection of an SRA domain in PvuRts1I strongly suggests that the protein flips the 5hmC or 5ghmC bases in its target sequence for detailed scrutiny, as suggested for UHRF1 (6–8) and SUVH5 (38) (based on crystallographic evidence) and for MspJI (10) (based on modeling). We first attempted to directly demonstrate nucleotide flipping using DNA with the environment sensitive fluorophore pyrrolocytosine (pyC) instead of the 5hmC in either one or both DNA strands. Preliminary experiments showed that pyC and 5hmC could both direct the cleavage of DNA oligoduplexes. With the 5hmC substrate (and a mixed 5hmC/pyC substrate) we observed two cleavage sites, one in the expected position and another one closer to the 5hmC base. With the pyC/pyC substrate, only the non-canonical cleavage closer to the base was observed (Supplementary Figure S4A). The pattern was not affected by the location of the histidine tag at either N- or C-terminus of PvuRts1I. Unfortunately, there was no significant increase of pyC fluorescence when PvuRts1I was added in the absence of divalent metal cations (Supplementary Figure S4B). This result is consistent with pyC (and by implication 5hmC or 5ghmC) not being flipped. However, it could also be due to the non-canonical cleavage for pyC substrates, to efficient quenching of the fluorescence by the SRA domain or to unintended tertiary structure of the oligoduplex (which eluted from a gel filtration column in several peaks).

As the pyC fluorescence experiments were inconclusive, we carried out mutagenesis experiments. According to the crystal structure Trp205, Trp215 and Glu228 might contribute to shaping the walls of the PvuRts1I pocket. In contrast, Arg208 contributes to the pocket primarily by its main chain, but not by the side chain, which points away from it. We separately changed all four residues to alanines. The Trp205Ala substitution made PvuRts1I insoluble, but the other variants could be assayed. The Arg208Ala mutation was only mildly compromised in its activity, but both the Trp215Ala and Glu228Ala mutations lost activity completely (Figure 3), as one would predict if the pocket of the SRA domain was required to accommodate the modified cytosine base. We conclude that the mutagenesis experiments support the hypothesis that the SRA domain of PvuRts1I flips the 5hmC or 5ghmC bases in a substrate.

## DISCUSSION

### A variant PD-(D/E)XK domain in PvuRts1I

The classification of PvuRts1I as a PD-(D/E)XK endonuclease is consistent with an earlier prediction (27) and supported by biochemical findings and the structural data. As noticed earlier and confirmed in this work, PvuRts1I is active in the presence of Mg^2+^, but not Ca^2+^ ions (Supplementary Figure S5). This is typical for PD-(D/E)XK restriction endonucleases, but would not be expected for HNH (also called ββα-Me), GIY-YIG or phospholipase like nucleases. The assignment is further supported by the presence of the core αβββ folding motif in the catalytic domain of PvuRts1I and by the presence of candidate active site residues (with the exception of the lysine) in their expected locations.

### Modification specific SRA domain, unspecific nuclease domain

PvuRts1I cleaves DNA approximately 11–13 nucleotides from 5hmC or 5ghmC bases. Taking the typical distance between adjacent base pairs as 3.4 Å (as in ideal B-DNA) and assuming straight DNA, the cleavage site is expected to be 30–45 Å away from the modified base. This distance is comparable to or larger than the largest linear dimension of the PD-(D/E)XK domain and therefore makes it unlikely that the nuclease domain is directly involved in sensing the DNA modifications. This suggests that the 5hmC or 5ghmC bases are ‘read’ by the SRA domain, in agreement with earlier findings for homologous domains of UHRF1 and MspJI, which are specific for modified DNA bases. Unfortunately, the isolated domains of PvuRts1I could only be expressed in insoluble form, and thus this model of PvuRts1I activity could not be directly tested biochemically.

### A model for PvuRts1I catalytic domain–DNA complex

**Figure 5.**
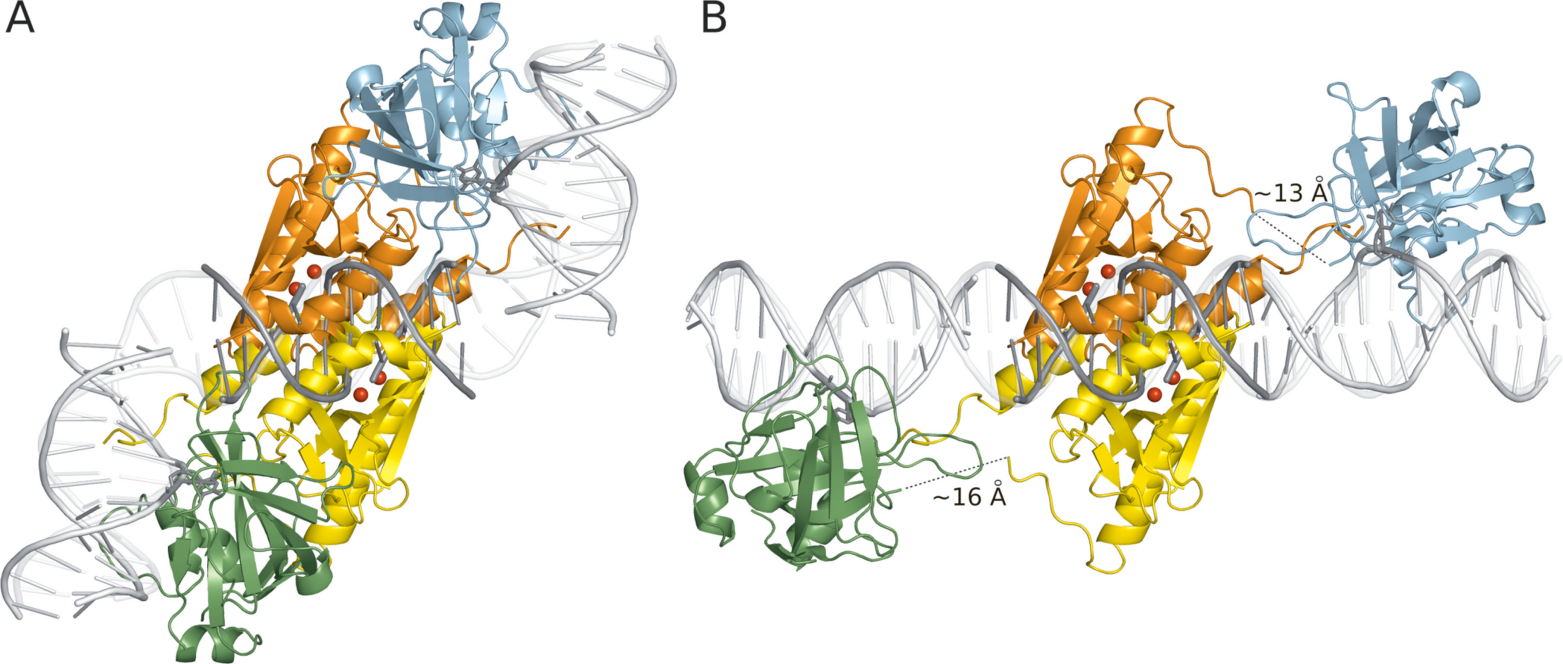
Models for the PvuRts1I dimer bound to target DNA. (A) Bent DNA and PvuRts1I with linker conformation observed in the crystal. (B) Straight DNA and PvuRts1I with adjusted interdomain linker. Nuclease domains (with modeled metal ions) were placed on the DNA based on the superposition with the NgoMIV-DNA complex (37). The binding mode of the DNA to the SRA domain is modeled after the UHRF1-DNA complex.

Based on prior co-crystal structures of PD-(D/E)XK domain restriction endonucleases with substrate DNA, such as the NgoMIV-DNA co-crystal structure (37), it is possible to place the PvuRts1I nuclease domain on the DNA in a ‘productive’ orientation (Figure 5). In fact, such modeling results in protein–DNA clashes only in the region of a single helix of the enzyme, which could move to the DNA major groove upon complex formation. The predicted DNA binding mode is also consistent with calculations of the surface properties of the protein, once the metal cations that are expected in the active site of a PD-(D/E)XK endonuclease (but were absent from the crystallization buffer) are included in the calculation (Supplementary Figure S6). As the stagger between the two single strand cuts is known (2-nt 3′-overhangs in the product) (25), the modeling also defines the relative orientation of the PD-(D/E)XK domains with respect to each other. In support of the model, resulting clashes between the nuclease domains appear resolvable by local rearrangements (Figure 5).

In agreement with the biochemical data for the PvuRts1I variant used in this work, we do not find the predicted dimer in the crystal. There is a crystallographic neighbor of the single molecule in the asymmetric unit in roughly the expected position, but its orientation is completely unlike what one would expect for a productive dimer. Moreover, the PISA server (39), which analyzes protein–protein contacts in a crystal, scores none of the interfaces in the PvuRts1I crystal as biologically relevant and classifies the protein as a monomer. The protein used in this work differs only by the N-terminal histidine tag from protein characterized previously (produced with an intein tag cleaved off during purification) (22). A comparison of the gel filtration patterns of N- and C-terminally tagged PvuRts1I suggests that the N-terminal tag slightly destabilizes the dimer. Thus, it appears that the tag, despite not being located in the dimerization interface, together with crystallization forces could be responsible for the unusual monomeric state of the protein in the crystal.

### A model for the full-length PvuRts1I dimer with bound DNA

Based on the co-crystal structure of the UHRF1 SRA domain with DNA (6–8), we can also model DNA bound to this domain of PvuRts1I. The next step then is to connect the DNA duplexes bound to the SRA and PD-(D/E)XK domains of the enzyme. Reassuringly, the biochemically predicted number of base pairs is very suitable to bridge the distance between the cleavage sites and the positions of the modified bases on the two DNA strands, provided that the DNA is sufficiently bent between the two regions (Figure 5A). Alternatively, a plausible model can also be built if PvuRts1I interdomain linkers are taken to be flexible and the domain orientations are adjusted so that the protein binds straight B-DNA (Figure 5B). We also note that the predicted DNA binding sites of both domains are qualitatively supported by calculations of the PvuRts1I electrostatic surface, which has clear patches of positive charge in these regions (Supplementary Figure S6). We presume that these parts of the protein are involved in interactions with the negatively charged phosphodiester backbone of DNA and account for the observed high unspecific DNA affinity.

The modeling data appear compatible with a FokI/TALEN-like (40) model for PvuRts1I activity (apart from the order of domains, catalytic domain is N-terminal in PvuRts1I and C-terminal in TALENs). According to this view, the SRA domains of the PvuRts1I dimer act as the modification specific counterparts of the sequence specific TAL domains, and the nuclease domains play the role of the FokI domains of the TALEN pair. The higher activity of PvuRts1I against substrates with two rather than one modified base could be an avidity effect, or might be attributed to an activating ‘kissing interaction’ between the nuclease domains (41).

## FUNDING

Foundation for Polish Science and the EU European Regional Development Fund [TEAM/2010-6/1 to M.B.]; National Science Centre [UMO-2011/02/A/NZ1/00052 to M.B.]. Funding for open access charge: TEAM/2010-6/1.

## SUPPLEMENTARY DATA

Supplementary Data are available at NAR Online.

SUPPLEMENTARY DATA
