# Supplementary material for: Crystal structure of the 5hmC specific endonuclease PvuRts1I
Source: Nucleic Acids Res. 2014 Mar 14;42(9):5929–36. doi: 10.1093/nar/gku186 (PMC4027163; doi:10.1093/nar/gku186)
Supplement: SUPPLEMENTARY DATA [file supp_42_9_5929__index.html]

Crystal structure of the 5hmC specific endonuclease PvuRts1I — Crystal structure of the 5hmC specific endonuclease PvuRts1I — SUPPLEMENTARY DATA 

# Crystal structure of the 5hmC specific endonuclease PvuRts1I

## SUPPLEMENTARY DATA

**Files in this Data Supplement:**

- SUPPLEMENTARY DATA
