# Supplementary material for: Crystal structure of the 5hmC specific endonuclease PvuRts1I
Source: Nucleic Acids Res. 2014 Mar 14;42(9):5929–36. doi: 10.1093/nar/gku186 (PMC4027163; doi:10.1093/nar/gku186)
Supplement: SUPPLEMENTARY DATA [file supp_gku186_nar-03690-h-2013-File007.pdf]

Crystal structure of the 5hmC specific endonuclease PvuRts1I

Asgar Abbas Kazrani[1*], Monika Kowalska[1*], Honorata Czapinska[1], Matthias Bochtler[1,2,$]

[1]*International Institute of Molecular and Cell Biology, Trojdena 4, 02-109 Warsaw, Poland*

[2]*Institute of Biochemistry and Biophysics PAS, Pawinskiego 5a, 02-106 Warsaw, Poland*

*Equal contribution

[§]Corresponding authors:

Tel: +48225970730; +48225970732

e-mail: mbochtler@iimcb.gov.pl

SUPPLEMENTARY MATERIAL

Supplementary Table

Supplementary Figures S1-6

Supplementary Methods

Supplementary Table:

Table S1: Data collection and refinement statistics

Data collection statistics	
Space group	*P* 4_1 2_1 2
a (Å)	62.0
c (Å)	211.0
Resolution range (Å)	50 - 2.35
Total reflections	100269
Unique reflections	17509
Completeness (%) (last shell)	96.6 (99.0)
I/σ (last shell)	19.5 (2.2)
R(sym) (%) (last shell)	4.8 (97.2)
B(iso) from Wilson ($Å^2$)	65.0
Refinement statistics	
Protein atoms excluding H	2499*
Solvent molecules	56
R_{cryst} (%)	20.3
R_{free} (%)	25.4
RMSD bond lengths (Å)	0.011
RMSD angles (°)	1.3
Ramachandran favored region (%)	97.3
Ramachandran allowed region (%)	100.0
Ramachandran disallowed region (%)	0.0

* Alternative conformations counted separately.

Supplementary Figures:

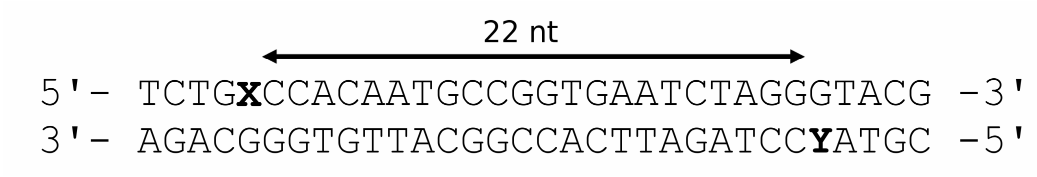

Figure S1: Oligoduplex used for DNA binding and cleavage experiments and for fluorescence studies. X and Y are the modified bases.

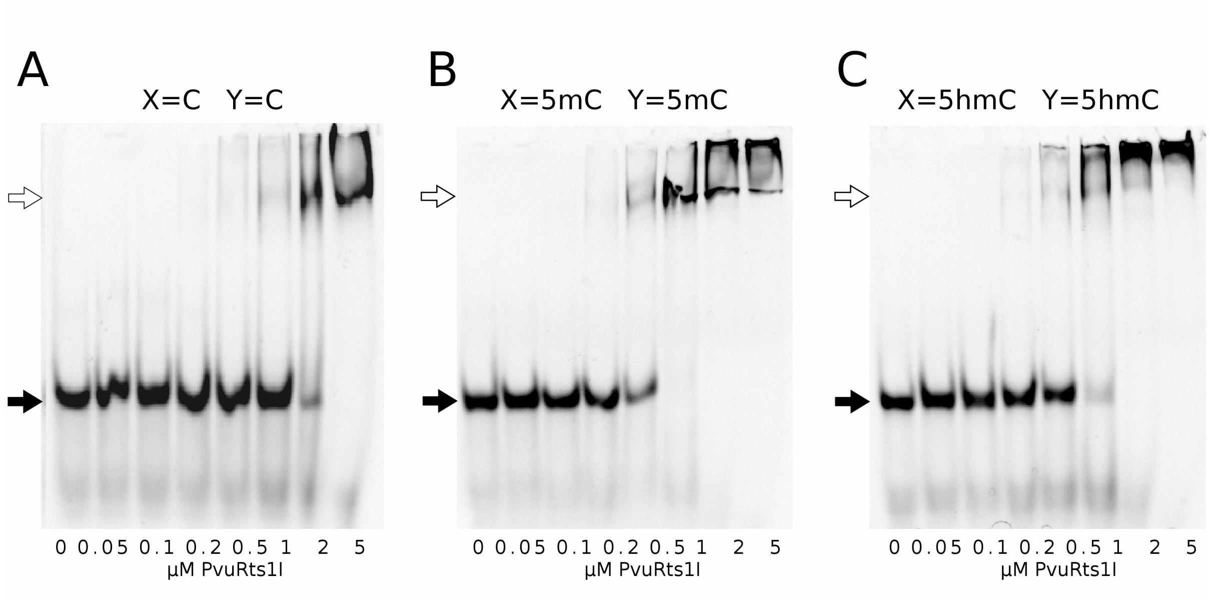

Figure S2: PvuRts1I binding to non-methylated (A), methylated (B) and hydroxymethylated (C) DNA with sequence as indicated in Fig. S1. The black arrows indicate unbound DNA substrate, the white arrows mark the protein DNA complex. 32-mer oligoduplex (2 µM, 20 pmols) and protein were incubated in 10 µl of buffer prior to electrophoresis.

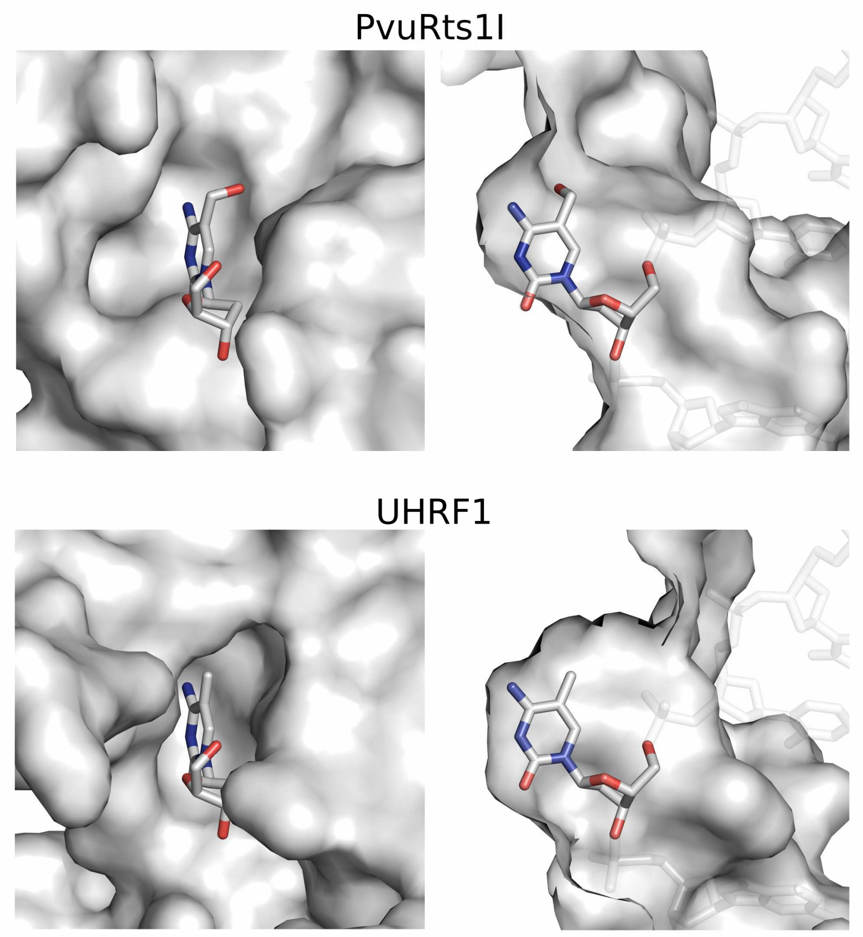

Figure S3: Space filling representation of the (candidate) binding pocket for the flipped base in PvuRts1I (top) and UHRF1 (bottom). Left and right panels differ by a 90° rotation around a vertical axis. In the right panels, a region of the protein has been removed to allow a view into the cavity. The modeled (PvuRts1I) or experimentally observed (UHRF1) modified base in the pocket is shown in stick representation. In the right panels, the rest of the DNA strand containing the flipped base is also displayed in very faint gray.

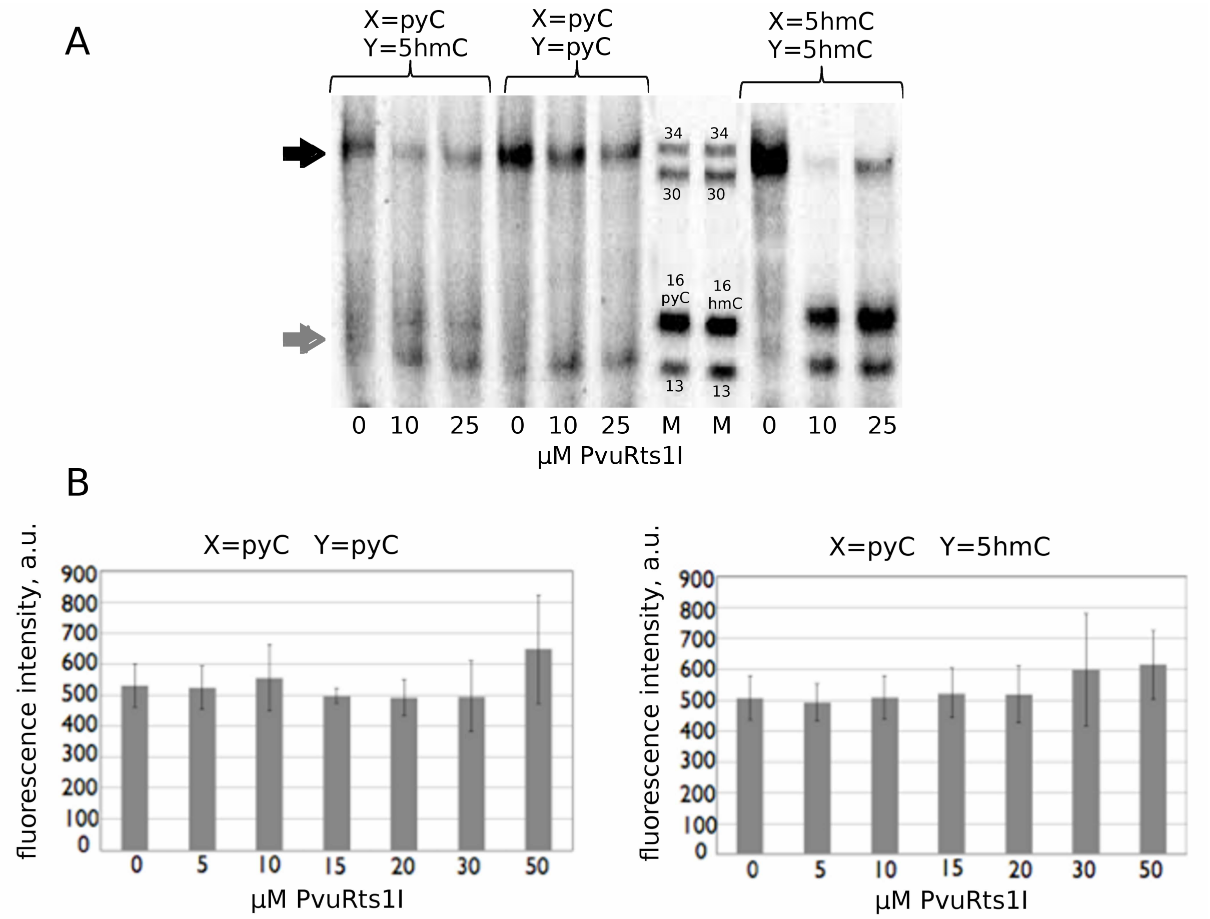

Figure S4: Fluorescence assay. (A) PvuRts1I activity against 0.5 µM oligoduplexes as indicated in Fig. S1. The 16-mer size standards contained the modified bases, but these did not affect migration. Substrates and standards were ^{33}P 5'-labeled, digested with PvuRts1I and analyzed by gel electrophoresis in a 20% urea gel. The black arrow marks the substrate, the grey arrow the cleavage product. (B) pyC fluorescence was excited at 350 nm and emission was quantified at 447 nm (the emission maximum in the buffer) in the presence of varying amounts of PvuRts1I.

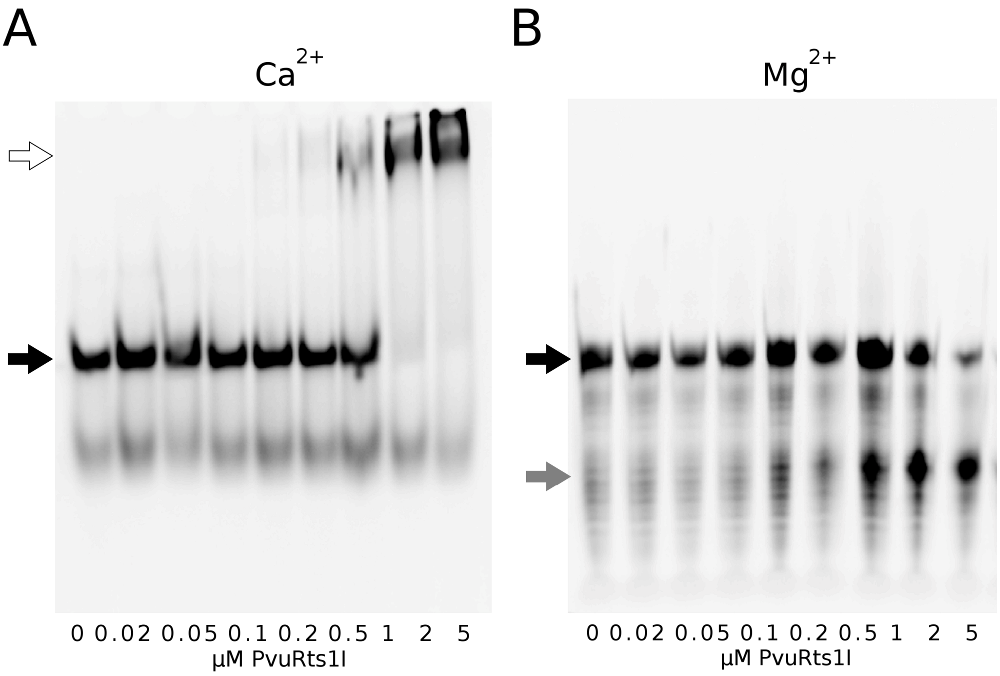

Figure S5: PvuRts1I activity assay in the presence of Ca^{2+} (A) and Mg^{2+} (B) ions. The white arrow indicates the protein DNA complex, the black arrows mark the unbound DNA substrate and the grey arrow denotes the DNA products after cleavage. 2 µM (20 pmols) of double-stranded 32-mer oligoduplex with 5hmC in both strands was used as a DNA substrate.

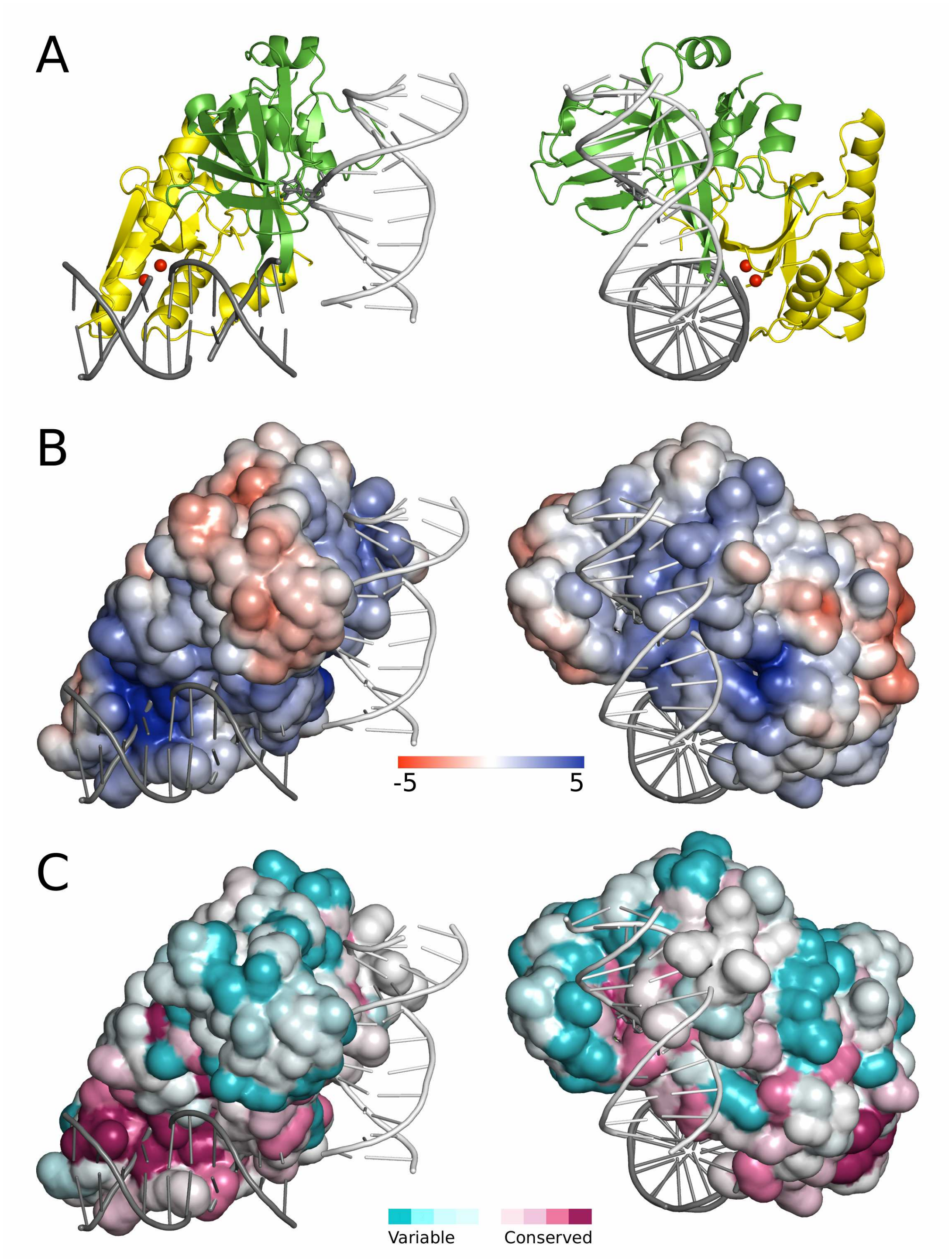

Figure S6: PvuRts1I monomer (in ribbon representation in panel A and surface representation in panel B) with modeled bound DNA molecules bound to catalytic and SRA domains (the DNA is not present in the crystals). In panel B, the protein surface is colored according to the electrostatic potential (in units of kT/e) calculated with the DelPhi program (Li et al. BMC Biophysics 2012, 5:9). In panel C, the protein surface is colored according to amino acid conservation in the PvuRts1I family as determined by the Consurf server (Celniker et al. Isr. J. Chem. 2013, 53:199). A helix that occupies the position of the DNA in the catalytic domain in the absence of DNA (residues 72-86) and that is likely to shift to the DNA major groove upon complex formation has been omitted from the figure.

Supplementary Methods:

Oligonucleotides: 5′-TCTG<u>*C*</u>CCACAATGCCGGTGAATCTAGGGTACG-3′ and 5′-CGTA<u>*C*</u>CCTAGATTCACCGGCATTGTGGGCAGA-3′ oligonucleotides either unlabeled or labeled with Cy5 on 3′ ends were purchased from Purimex (Germany) (<u>*C*</u> stands for unmodified cytosine (C), 5-methylcytosine (5mC), 5-hydroxymethylcytosine (5hmC), or pyrrolocytosine (pyC)). They were dissolved in 20 mM Tris/HCl pH 8.0 and annealed by heating up to 95 °C followed by slow cooling to 4 °C to yield eight combinations of DNA duplexes: C-C, 5mC-C, 5hmC-C, 5mC-5mC, 5hmC-5hmC, 5hmC-5mC, pyC-5hmC and pyC-pyC.

Fluorescence experiments: Fluorescence experiments were carried out using a Shimadzu RF5301 fluorescence spectrometer (3 nm emission and excitation slits) using 10 μM oligoduplexes with either 5hmC and pyC in the two strands or pyC in both strands and varying amounts of protein in buffer C (20 mM Tris/HCl pH 7.6, 200 mM NaCl, 1 mM EDTA and 1 mM DTT) at 25°C. Fluorescence was excited at 350 nm, and emission was quantified at 447 nm (the emission maximum). Fluorescence of the protein preparation alone (probably due to a contamination) was subtracted from the reading for protein DNA complex. All measurements were carried out in triplicate for determination of standard errors.

Analytical gel filtration: 50 μg (1.4 nmol) of PvuRts1I protein was separately mixed with blunt-ended 32-mer C-C, 5hmC-C and 5hmC-5hmC oligoduplexes in buffer D (20 mM Tris/HCl pH 7.6, 200 mM NaCl, 5 mM $CaCl_2$ and 1 mM DTT) at different stoichiometric ratios, and incubated on ice for 1 h. The samples were loaded on a Superdex 75 HR 10/300 column (GE Healthcare), which was equilibrated in buffer D and run with a flow rate of 0.4 ml/min. The column was calibrated with Bio-Rad protein standards (vitamin B-12, 1.35 kDa; myoglobin, 17 kDa; ovalbumin, 44 kDa; IgG, 150 kDa and thyroglobin, 670 kDa). Elution profiles were monitored by an Ettan LC System (GE Amersham) two-wavelength detector at 260 and 280 nm. For the interpolation of unknown molecular mass, a linear dependence of the logarithm of the molecular mass on the elution time was assumed.

Gel mobility shift assay: 3′-Cy5-labeled oligoduplexes at 2 μM concentration were mixed in the PvuRts1I binding buffer (20 mM Tris/HCl pH 8.0, 150 mM NaCl, 5 mM $CaCl_2$, 0.1 mg/ml BSA) with increasing amounts of protein (0-5 μM), incubated for 1 h at room temperature. The 10 μl reaction mixtures were combined with 6×DNA loading dye solution

(10 mM Tris/HCl pH 7.6, 0.03% bromophenol blue, 0.03% xylene cyanol FF, 60% glycerol, 60 mM EDTA) and analyzed on the nondenaturing 8% polyacrylamide gel using 89 mM Tris/borate pH 8.3, 5 mM $CaCl_2$ as running buffer. Cy5-labeled DNA was detected by ImageQuant LAS 4000 system (GE Healthcare).

DNA cleavage assays: The PvuRts1I cleavage reactions were performed at 37 °C by mixing either Cy5- or ^{33}P-labeled oligoduplexes (2 μM and 0.5 μM respectively) with increasing amounts of protein (0-25 μM) in 10 μl reaction buffer (150 mM NaCl, 20 mM Tris/HCl pH 8.0, 10 mM $MgCl_2$, 1 mM DTT). After 1 h the reactions were quenched with 2×formamide loading dye solution (98 % v/v formamide, 10 mM EDTA, 0.1% bromophenol blue). Separation of DNA hydrolysis products was performed by denaturing PAGE in Tris/borate containing 7.0 M of urea was run at 30 V/cm. Cy5-labeled DNA was detected by ImageQuant LAS 4000 system (GE Healthcare). The radiolabeled DNA was detected by Typhoon TRIO (GE Healthcare).
